# Early maturation of sound duration processing in the infant’s brain

**DOI:** 10.1038/s41598-023-36794-x

**Published:** 2023-06-24

**Authors:** Silvia Polver, Gábor P. Háden, Hermann Bulf, István Winkler, Brigitta Tóth

**Affiliations:** 1 Department of Psychology, University of Milano-Bicocca, Milan, Italy; 2 Institute of Cognitive Neuroscience and Psychology, Research Center for Natural Sciences, Budapest, Hungary; 3 Department of Telecommunications and Media Informatics, Faculty of Electrical Engineering and Informatics, Budapest University of Technology and Economics, Budapest, Hungary; 4 NeuroMI, Milan Center for Neuroscience, University of Milano-Bicocca, Milan, Italy

**Keywords:** Cortex, Perception

## Abstract

The ability to process sound duration is crucial already at a very early age for laying the foundation for the main functions of auditory perception, such as object perception and music and language acquisition. With the availability of age-appropriate structural anatomical templates, we can reconstruct EEG source activity with much-improved reliability. The current study capitalized on this possibility by reconstructing the sources of event-related potential (ERP) waveforms sensitive to sound duration in 4- and 9-month-old infants. Infants were presented with short (200 ms) and long (300 ms) sounds equiprobable delivered in random order. Two temporally separate ERP waveforms were found to be modulated by sound duration. Generators of these waveforms were mainly located in the primary and secondary auditory areas and other language-related regions. The results show marked developmental changes between 4 and 9 months, partly reflected by scalp-recorded ERPs, but appearing in the underlying generators in a far more nuanced way. The results also confirm the feasibility of the application of anatomical templates in developmental populations.

## Introduction

One of the main characteristics of auditory information is that sound unfolds in time. Therefore, the integration of information across time is crucial for perception on multiple timescales^1^. Infants are sensitive to a number of temporal sound features at birth, such as gaps, sound onset and offset, and changes in sound duration^2–4^. Thus, these abilities are thought to be innate and to support the foundation for language acquisition^2,5,6^. Similarly, to adults, infants utilize temporal cues, such as phoneme duration, for segmenting sound sequences^7^, including finding words within continuous speech^5^. Therefore, the ability to process rapidly changing sounds has been suggested to be a critical skill for language development^8,9^. Indeed, the ability to quickly perform fine-grained acoustic analyses is critical for speech decoding^10,11^. Further, neurophysiological findings suggest that neurons in primary and closely related auditory regions exhibit complex spectro-temporal receptive fields, participating in the encoding of auditory features at multiple timescales^12,13^. However, despite their importance, the mechanisms underlying the processing of auditory temporal features are still poorly understood. The present study aimed to investigate the early development of the processing of an important auditory temporal feature: sound duration. To this end, auditory event-related brain potentials (ERP) and their neural generators elicited by tones and noises of different duration were compared between four and 9-month-old infants.

ERPs have been long used to study the neural substrate of auditory temporal processing and their development in infancy^2,3,14^. Specifically, some previous ERP studies have shown that the newborn brain is sensitive to sound duration, and it detects unexpected changes in the duration of a repeating sound^3,15,16^^.^ The adult ERP components sensitive to sound duration comprise the P1-N1-P2-N2 complex^3^. The adult P1–N1–P2–N2 sequence begins at around 40–50 ms after stimulus onset and lasts until ca. 150–250 ms^17^^.^ Similar waveforms have been observed in newborns, infants, and in 5 to 10 years old children^2^. However, it is debated whether the underlying functional mechanisms and brain areas involved are the same as those observed in adults^18^. Indeed, several maturational mechanisms, such as advancing myelination and increasing synaptic connections are at play across development that can affect ERPs^19^.

Generally, neonatal ERPs exhibit a large positive deflection at midline starting at about 100 ms and ending 450 ms from stimulus onset, followed by a low-amplitude negative deflection at approximately 450–600 ms (N450). These waveforms are thought to reflect precursors of the P2 and N2^20^. At about 3 months of age, the P2 is reportedly split by the appearance of a negative deflection, and between three and 6 months, another deflection peaking at about 250–280 ms (N250) appears. The two negative waveforms are assumed to represent precursors of the adult N1 and N2, respectively^20^. By 6- to 9 months of age the separation between the N1 and the N2 precursor waveforms becomes well-defined, making the infantile ERPs’ morphology resemble the P1–N1–P2–N2 complex^18,21,22^. The developmental trajectories of ERP waveforms point toward the presence of underlying developmental milestones from three to 9 months of age^18,21,22^. Unfortunately, studies directly addressing changes along development are still scarce.

However, scalp recordings do not directly reflect the underlying generators. Sound perception activates multiple pathways in the brain. As a result, scalp-recorded waveforms may represent summed activity from several generators^23,24^. Moreover, different ERP generators may have different maturational courses^20^. Therefore, discerning and locating these generators and addressing their developmental trajectory provides important information for anchoring functional changes in brain maturation and plasticity. In adults, generators of the P1–N1–P2–N2 complex have been found to reside in the primary auditory cortex (Heschl’s gyrus), the superior temporal gyrus (STG), in thalamo-reticular systems, in the planum temporale (encompassing Wernicke's region), and in supratemporal auditory cortices^23,25–27^. Regarding developmental populations, source analysis revealed that presenting syllables to 6-month-old infants bilaterally activated auditory and frontal cortical areas, and the anterior cingulate cortex^28^. Six-months-old infants also show activity in supratemporal and frontal areas in response to sound discrimination^29^. However, until recently, the lack of age-appropriate templates has complicated the application of source localization in infants, because the quality of age-appropriate head models determines the accuracy of source localization^24^. Here we rely on the recently released infant templates^30^ derived from the Neurodevelopmental MRI Database^31^ and made ready for use by their implementation in the Brainstorm software^32^.

We investigated duration-sensitive auditory ERP waveforms and their sources to establish their developmental changes between 4 and 9 months of age. We chose these two age groups because they bracket important milestones in the development of ERP morphology (see above). Infants were presented with four types of sound differing in duration (200 vs. 300 ms) and sound quality (harmonic tones vs. white noise). We expected to find significant changes in ERP morphology between the two age groups in accordance with previous descriptions of ERP development. Indeed, in contrast to the simpler ERP component structure at 4 months, by 9 months of age, we expected to find precursors of the adult ERP complex in the form of a positive deflection, accompanied by two negative ones coinciding with the N250 and the N450 latencies^20^. We then asked whether either or both waveforms show sensitivity to sound duration and whether sensitivity to sound duration is dependent on the nature of the sound (harmonic tones vs. noise segments). To avoid confounds related to biased choices of time points and electrodes of interest we referred to the cluster-based statistic^33^. In this way, we were able to cluster activity over time and space.

We expected to find the main sources of duration-sensitive activity in brain areas belonging to the auditory brain network, including the primary auditory cortex (PAC), and activity in network areas previously shown to be sensitive to sound duration, such as the STG and inferior frontal areas^34^. Further, in line with our expectations for scalp-recorded ERPs, we hypothesized that 9-months-old infants would show stronger activity in a more distributed network comprising fronto-central areas compared to 4-months-old infants in the time window(s) of the effect(s) found over the scalp. However, to control for multiple comparison issues we again chose to refer to a data-driven approach in the form of non-parametric permutation testing.

## Results

**Figure 1:**
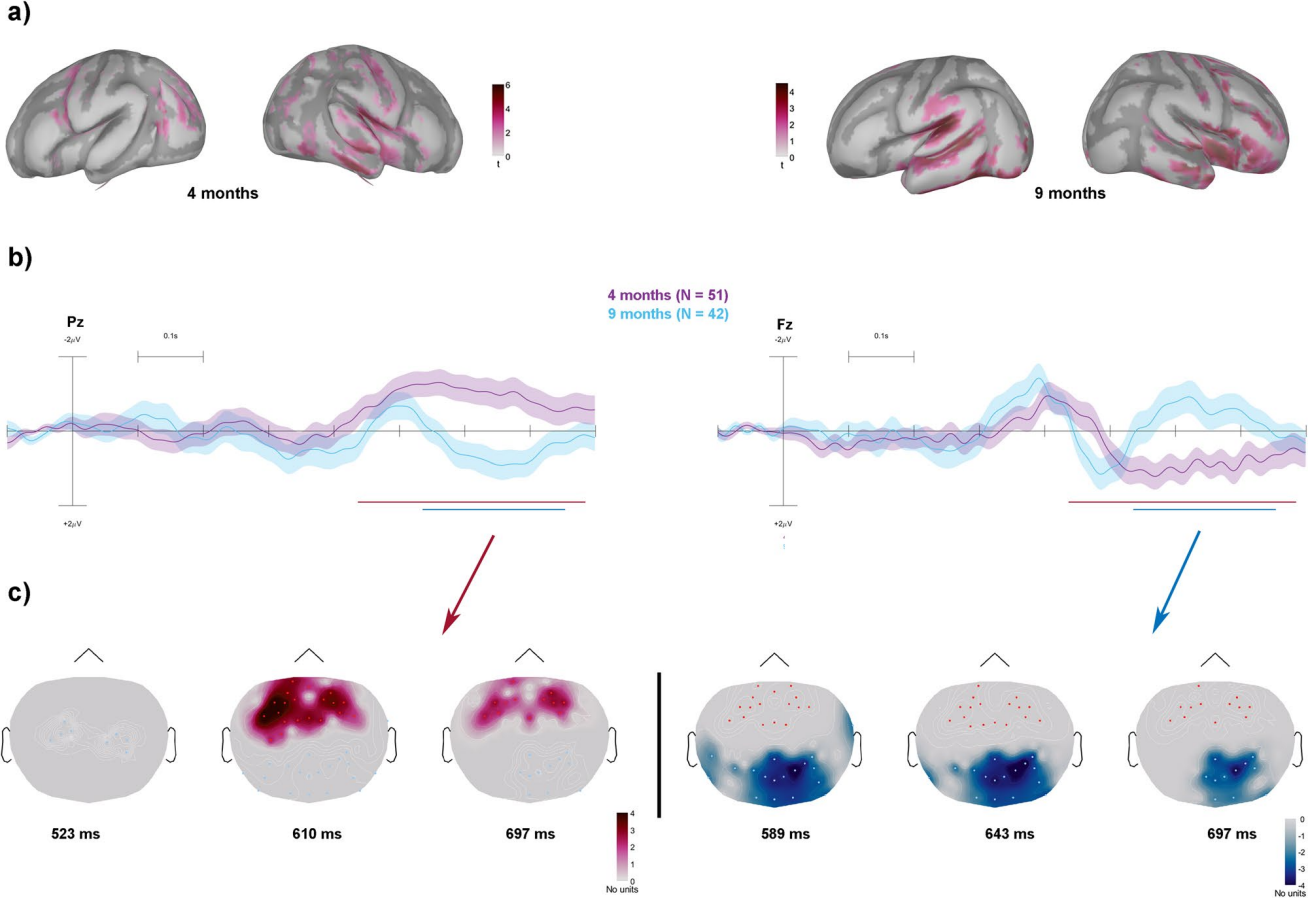
(**A**) Significant current source density difference between the responses to long (300 ms) and short (200 ms) tones after permutation testing depicted over the inflated cortex. Source activity plotted via Brainstorm (version 2022 February; https://neuroimage.usc.edu/brainstorm) (**B**) Grand-average ERP difference waveforms (300–200 ms) elicited by tones for 4- and 9-month-olds (marked by line colors) from a parietal (Pz, left) and a frontal lead (F4, right). The time windows of clusters are drawn with solid lines below the ERPs. Scalp activity plotted via EEGLAB software (std_plotcurve function; https://www.mathworks.com/matlabcentral/fileexchange/56415-eeglab) (**C**) Topographical distribution across the scalp of the clusters differentiating the two age groups. The positive cluster indexes stronger activity at 4- compared to 9-months, while the negative cluster indexes stronger activity at 9- compared to 4-months. Topographical maps are plotted via Brainstorm toolbox.

**Figure 2:**
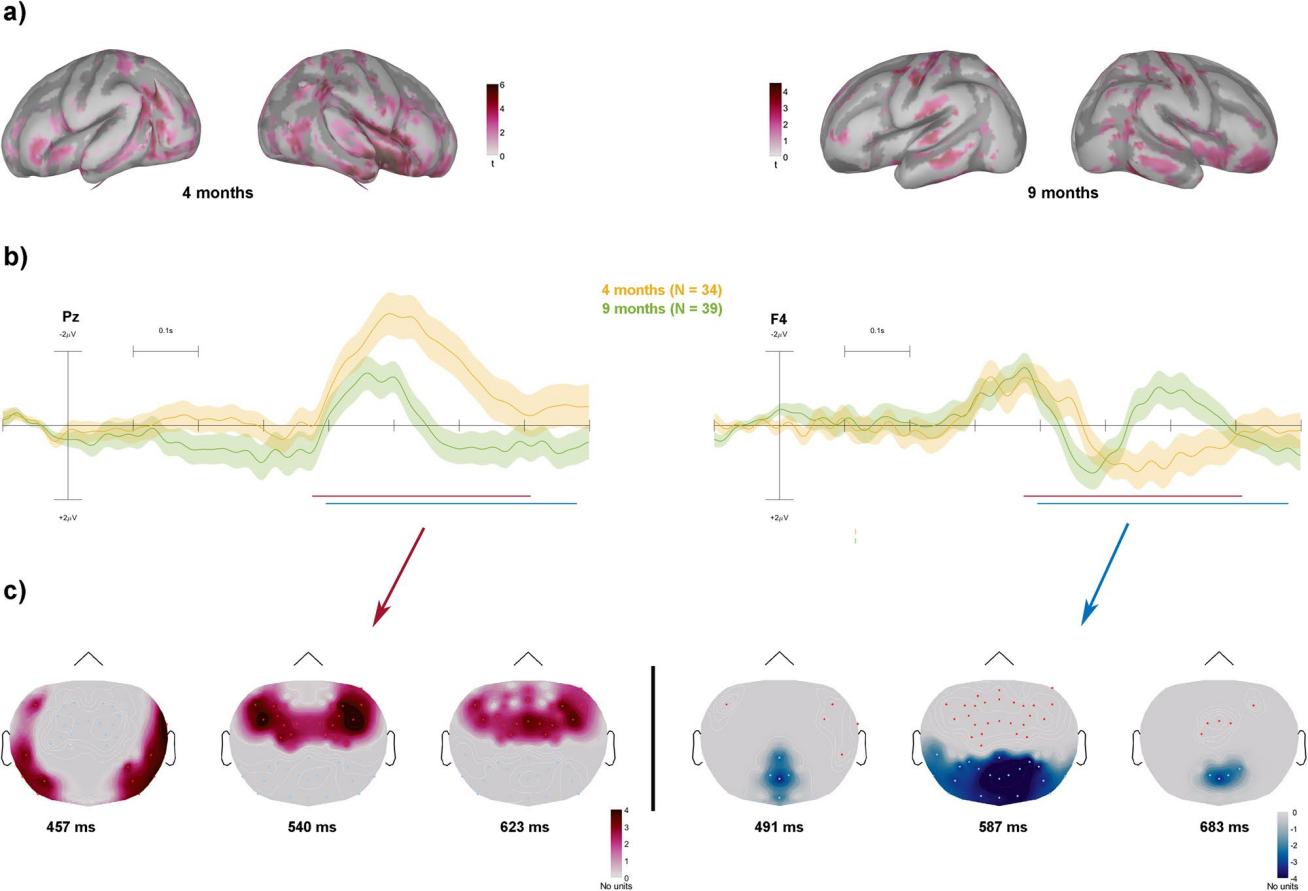
(**A**) Significant current source density difference between the responses to long (300 ms) and short (200 ms) noise segments after permutation testing depicted over the inflated cortex. Source activity plotted via Brainstorm (version 2022 February; https://neuroimage.usc.edu/brainstorm) (**B**) Grand-average ERP difference waveforms (300–200 ms) elicited by noise segments for 4- and nine-month-olds (marked by line colors) from a parietal (Pz, left) and a frontal lead (F4, right). The time windows of clusters are drawn with solid lines below the ERPs. Scalp activity plotted via EEGLAB software (std_plotcurve function; https://www.mathworks.com/matlabcentral/fileexchange/56415-eeglab ) (**C**) Topographical distribution across the scalp of the clusters differentiating the two age groups. The positive cluster indexes stronger activity at 4- compared to 9-months, while the negative cluster indexes stronger activity at 9- compared to 4-months. Topographical maps are plotted via Brainstorm toolbox.

We performed a cluster-based permutation test on the scalp topographies, to investigate the spatial distribution and timing of the developmental differences in duration-sensitive neural activity, separately for tone (Fig. 1) and noise sounds (Fig. 2). The difference waveforms between long (300 ms) and short (200 ms) sounds were tested for the presence of the previously described N250 and the N450 components. The cluster-based statistic revealed age effects (*p* < 0.05) in overlapping positive (4 months > 9 months) and negative (9 months > 4 months) clusters in the 400 to 800 ms latency range for tones (Fig. 1C) and noise segments (Fig. 2C). The grand-average difference waveforms (long–short stimuli) of 4- and 9-months-old infants are shown in Figs. 1 and 2 (panels B).

Specifically, for tones (Fig. 1), we observed differential activity across age groups in a positive cluster (*t*_*sum*_ = 7622, *p* = 0.018, *95% CI* = 0.0082) comprising higher fronto-central activity at 4 months, compared to 9 months from 536 to 753 ms post-stimulus. We also observed a negative cluster (*t*_*sum*_ = − 11,844, *p* = 0.002, *95% CI* = 0.0028) comprising higher activity over posterior electrodes for 9-month-olds, compared to 4-month-olds from 437 to 784 ms.

Based on age-appropriate template brain anatomies and default electrode locations, we then inferred the activity on the cortical surface. The tone-evoked source activity (average between 400 and 550 ms) for long and short tones was entered into permutation testing (*p* < 0.05) separately for 4- and 9-month-olds. In the 4-month-olds, the neural generators significantly sensitive to tone duration differences were focused on the left temporal gyrus (including the PAC; see Fig. 1, panel A top left) and the precentral gyrus (premotor area). In the 9-month-olds, significant tone duration sensitivity was observed bilaterally in the temporal gyrus (including the PAC see Fig. 1, panel A top right) and in the prefrontal gyrus.

For noise segments (Fig. 2), we found a positive cluster differentiating 4 and 9-month-olds (*t*_*sum*_ = 12,508, *p* = 0.008, *95% CI* = 0.0055) comprising higher activity over lateral and fronto-central electrodes for 4- compared to 9-month-old between 375 and 709 ms. We also found a negative cluster (*t*_*sum*_ = 12,242, *p* = 0.006, *95% CI* = 0.0048) indicating that the noise segments elicited higher neural activity over central and posterior leads in the 9- compared to 4-months-olds in a time window starting at 396 ms and ending at 780 ms.

The noise-evoked source activity (average between 400 to 550 ms) for long and short tones was entered into permutation testing (*p* < 0.05) separately for 4 and 9-month-olds. For the 4 months old group the significant duration sensitive source activity was distributed but most dominantly located in the left temporal gyrus (including PAC, Fig. 2 panel A top left) and the prefrontal cortices. For the 9-month-old group, the sound duration sensitive responses were again localized bilaterally in temporal areas with further activity in the premotor and prefrontal areas (Fig. 2 panel A, top right).

## Discussion

Results of the current study showed that the brain of both 4- and 9-months-old infants is sensitive to sound duration, as reflected by ERPs elicited by short, isolated sounds. Effects of maturation were found for the scalp-recorded waveforms and the corresponding source activity. Both scalp-recorded ERPs and the related source activity became spatially more circumscribed between 4 and 9 months of age. Generators of the event-related potentials sensitive to sound duration were located mainly in the ventral fronto-temporal auditory pathway coupled with activity in other key regions involved in language processing. Details of the results are discussed below.

We found that the responses of 4 and 9-months-old infants differed within a relatively long time window (from ~ 400 to 780 ms) for both tones and noise segments. Our ERP results are generally congruent with reports of waveforms related to the N250 and N450 in infant studies with duration manipulation^3,20,35,36^. While we did observe longer latencies in younger infants in the present study compared to some of the previous ones, this may be due to variability in the maturational stages of auditory brain networks^37^. Indeed, while latencies remained approximately the same across age groups, we observed that posterior activity differentiated 9 from 4 months. Although this result contrasts findings of a developmental shift toward frontal regions of auditory ERPs^6^, other reports point toward a nonlinear developmental trajectory of ERPs scalp topography^38^. Indeed, different generators underlying the same ERP can have different maturational pathways^37^. Thus, the contrasting age-related differences in generator activity may be related to differential maturational trajectories^39^. This hypothesis is strengthened by previous reports highlighting that ERP components underlying duration perception are not unitary in nature and reflect dynamic processes involving multiple brain regions^40^. Interestingly, the spatial extent of the clusters differentiating the responses of 9- and 4-months-old infants more closely reflect the actual spatial locations of temporal generators we observed, compared to the frontal activities described in the literature^41^.

The source-localized results show that the generators underlying the responses to tones at 4 months were mainly located in the left temporal gyrus comprising the PAC and in sensorimotor cortices (see Fig. 1A). The neural generators in response to noise segments at 4 months were located in overlapping areas with the addition of the prefrontal cortices (see Fig. 2A). At 9 months, in response to tones, generators were mainly located bilaterally in the temporal gyrus and in the prefrontal gyrus. In response to noise segments, at 9 months, we again observed bilateral activity in temporal cortices, coupled with activity in premotor areas and in prefrontal cortices. From the source maps one can discern more focal distribution within primary and secondary auditory areas at 9 compared to 4 months of age. The duration-sensitive sources found in this study are consistent with those reported in the literature in response to both speech sounds and tones^26,37,42–45^. Moreover, we observed the involvement of sensorimotor cortices confirming reports of a central role of sensorimotor networks in duration perception^46,47^. This points towards the reliability of the use of anatomical templates in developmental samples^48,49^, which represents an important turning point in studying electrical brain activity in infants. A somewhat surprising result was the similarity between the scalp-recorded ERP morphologies of 4- and 9-months-old infants. Because the infants' brain is subject to dramatic development during this period^37^, one could have expected the morphology of all ERP responses to change accordingly. However, the similarity between the results in the two age groups sheds light on the central role that the processing of temporal information plays in sound processing: the processing of temporal features is more consistent than that of spectral features throughout development and even its neural substrate may not change dramatically from infancy to adulthood. Thus, whereas pitch discrimination initially requires a very large separation between the sounds to elicit reliable discriminative brain activity^50^, temporal features are reliably detected already at birth^4,14^ and responses sensitive to different sound durations are quite similar between neonates and adults^3^.

The underlying generators show a more nuanced picture. The cortical distribution of the sound duration-sensitive generators is almost identical in the two age groups (Figs. 1 and 2). The exception is represented by a narrowing of activity near primary and secondary auditory cortices, suggesting the presence of brain maturational mechanisms. Supporting this assumption, we found statistically significant increments in current source density in the right hemispheric part of the previously described fronto-temporal network. The narrowing of activity we observed may thus be based on the higher efficiency of the primary and secondary auditory sensory cortices.

Age-related effects on duration-sensitive ERPs and their generators may stem from multiple types of maturational processes^20^. For example, myelination of auditory pathways plays a crucial role by enabling rapid coordination among neurons^51^. The timeline of myelination in auditory pathways mirrors key developmental milestones in auditory perception such as the appearance of the P1–N1–P2–N2^51^. Regarding the involvement of frontal regions, despite the widely accepted notion that prefrontal areas show prolonged development compared to sensory cortical regions^39^, some studies show that already at birth infants recruit frontal areas during processing speech sound^52–54^. Moreover, temporal and frontal regions do not develop independently but show high correlations in their developmental course^55^. These findings suggest that the frontal cortices and their connections to auditory sensory regions (temporo-parietal areas) play a major role in language acquisition. In infants, frontal and temporo-parietal regions are already well connected, and these connections are hypothesized to underlie the early processing of speech^56^. Both bottom-up and top-down connections are necessary for language^57^. We hypothesize that the duration-sensitive component we observe may reflect a first prodromal involvement of frontal cortices in auditory temporal processing (also related to language acquisition) feeding back from temporal areas. In this sense, this component may reflect a maturational step of duration processing which might not yet be stable at 4 months.

The current results provide new information on the development of the processing of an important temporal parameter, sound duration. The ERP results are generally compatible with those from previous studies. The more reliable source reconstruction offered by the recently released infant structural anatomical templates^30^ provided new insights into the generators underlying duration processing and their development, allowing one to speculate about the functional and developmental significance of this neural activity. Naturally, these initial hypotheses require further studies for confirmation and specification.

## Methods

### Participants

Infants were recruited as part of a broader longitudinal study aimed to assess the role of infant-directed speech in speech acquisition. In this manuscript, we will refer only to two separate age cohorts: the first one comprised 64 (20 males) 4 months old, and the second cohort 63 (19 males) 9 months old healthy infants. All infants were firstborns, none of them twins. The average age at the recording time was 4.22 months (*SD* = 0.2) for the 4-months-old and 9.52 for the 9-months-old group (*SD* = 0.36). Due to the longitudinal nature of the study, 13 of the infants in the final sample were tested at both 4 and 9 months.

Recordings were carried out at the sound-attenuated infant laboratory of the Institute of Cognitive Neuroscience and Psychology, Research Centre for Natural Sciences, Budapest, Hungary. Informed consent was obtained from one (mother) or both parents. The study was conducted in full accordance with the Declaration of Helsinki and all applicable national and international laws, and it was approved by the United Ethical Review Committee for Research in Psychology (EPKEB), Hungary.

### Stimuli and procedure

All infants were presented with shorter (200 ms) or longer (300 ms) sounds grouped into two separate blocks each consisting of 150 short and 150 long sounds delivered in random order. One of the stimulus blocks presented tones of 500 Hz base frequency with 3 harmonics of 50, 33, and 25 percent amplitude, respectively, summed linearly together (‘tone’ condition). The other stimulus block comprised frozen white noise segments (‘noise’ condition). All sounds were presented at approximately 70 dB SPL loudness and the sounds were attenuated by 5 ms long raised-cosine onset and offset ramps. The onset-to-onset interval was 800 ms. The tone condition was always delivered first, and there was a short rest of 30–60 s between conditions. Stimuli were created and delivered using Matlab (MathWorks Inc., Natick, MA, USA) and Psychtoolbox^58^ software. The sound signal from the computer was amplified by a Yamaha A-S301 amplifier (Hamamatsu, Japan) and presented through a pair of speakers (Boston Acoustics A25, Woburn, MA, USA). The speakers were positioned ca. 1.75 m in front of the participant, 70 cm apart from each other.

The infant sat comfortably in her mother's lap while the experimenter employed toys to keep the infant facing toward the loudspeakers and his/her attention away from the electrode net. The mother was listening to music through closed-can audiometric headphones to isolate her from the experimental stimulation. If the infant became fussy, the playback was stopped, and the experimenter attempted pacification. If pacifying was successful the playback restarted from the beginning, otherwise, the experiment was discontinued. The experiment reported here was presented first within a session combining multiple experiments. Data from the other experiments and the associations across results will be published separately after collecting the complete data set. As the current experiment was always the first one presented, the other experiments are not listed.

### EEG data acquisition and preprocessing

EEG was recorded with a 60-electrodes HydroCel GSN net (64 electrodes v1.0 layout, geodesic; electrodes 61–64 were connected to the ground in the small pediatric caps used) and a GES 400 DC amplifier passing the digitized signal to a computer running the NETSTATION v5.4.1.1 software (both Electrical Geodesics, Eugene, OR, USA). Signals were recorded online at a sampling rate of 500 Hz with the Cz reference (DC, no online filtering). Electrode impedance during recording was kept below 50 kΩ.

The recorded signals were imported to MATLAB (Mathworks, Natick, MA; ver. 2021a) and processed using EEGLAB^59^ through the HAPPE + ER pipeline validated for developmental samples^60^. First, line noise was reduced through the multi-taper regression implemented in the CleanLine plugin^61^. Then an automatic low-pass finite impulse response (FIR) filter of 100 Hz was applied to the data. Electrodes were removed if non-functioning for longer than 5 s, containing outliers (> 3.5 standard deviations or < − 5 standard deviations from mean power), affected by line noise (> 6 standard deviations from mean line noise/neural signal ratio), and their correlation with other electrodes was < 0.8. Data were then subjected to soft wavelet-thresholding correction. Afterward, data were band-pass filtered between 0.1 Hz and 30 Hz using a finite impulse response (FIR) filter (Kaiser windowed, Kaiser β = 5.65, filter length 4530 points). From the continuous EEG records, epochs were extracted between − 100 and 800 ms relative to the sound onset, separately for the two STIMULUS DURATIONs (short- and long-duration sounds), and STIMULUS TYPEs (tones and noises). Baseline correction was then applied referring to the -100 to 0 ms window. Electrodes were deemed noisy and rejected if their joint probability exceeded by 3 *SD* the mean of activity of all other electrodes. Afterward, electrodes were interpolated, and data were re-referenced to the average reference. A threshold of +/− 150 µV was set to reject epochs with abrupt amplitude changes, while a joint probability-based rejection was again used to remove epochs exceeding by 3 *SD* the mean of overall activity. Indeed, joint probability-based rejection is useful in isolating high-frequency artifacts such as muscle activity^60^. Infants’ data was not used if the remaining epochs for each STIMULUS DURATION were under 70 for either STIMULUS TYPE, or if the number of the rejected electrodes per subject was greater or equal to 10. Data recorded from 13 four-month-old infants and 10 nine-month-old infants were excluded due to excessive artifacts. Because some infants did not complete both stimulus blocks after artifact rejection 51 four-months-old and 42 nine-months-old infants’ data were analyzed for the tone condition, and 34 four-months-old and 39 nine-months-old infants’ data for the noise condition. As only 13 infants were tested at 4 and 9 months, we did not investigate longitudinal effects in this study.

Epochs of each remaining infant were averaged separately for each STIMULUS DURATION, STIMULUS TYPE, and AGE GROUP. The mean number of epochs is reported in Table 1. No significant differences were found for average epoch numbers between conditions or groups.

**Table 1:** The mean number of epochs remaining after preprocessing was reported separately for STIMULUS DURATION, STIMULUS TYPE, and AGE GROUP.

	Short tones	Long tones	Short noises	Long noises
four-months-old	116.82 (SD = 11.63)	117.11 (SD = 13.87)	105.28 (SD = 13.82)	104.79 (SD = 13.45)
nine-months-old	108.17 (SD = 18.42)	107.68 (SD = 18.34)	112.75 (SD = 22.31)	111.91 (SD = 21.79)

### ERP analysis

First, we calculated the difference waveforms by subtracting the average ERP elicited by short stimuli from the average ERP elicited by the corresponding long ones. Then to compare the age groups, we applied the cluster-based statistic to avoid biased choices of time points and electrodes of interest while investigating scalp ERPs ^33^. Specifically for every sample (i.e., each electrode and time point across averaged epochs), a *t-value* was calculated between the two age groups, thus creating pairwise group contrast matrices. Selected samples were then clustered in connected sets, on the basis of temporal and spatial adjacency. Cluster-level statistics were calculated by taking the sum of the *t-values* for every cluster. The significance probability for the cluster-based statistic was then calculated via the Monte Carlo method with a 0.05 *p* cut-off. More in-depth, subjects belonging to different groups were randomly sorted and the test statistic was calculated on these random partitions. The number of random permutations needed to construct the histogram of the test statistics was set to 1000. Within this framework, *p-values* were the proportion of random partitions that resulted in a larger statistic than the observed one. A *p-value* was extracted for each cluster. This process was separately done for the tone and the noise condition. All steps were completed using the Brainstorm toolbox (version 2022 February)^32^.

### Source localization

The Brainstorm toolbox was also used to perform EEG source reconstruction, following the protocol of previous studies^62^. The noise covariance matrix was computed over the baseline and block by block, in order to avoid effects caused by slow drifts in the data. Age-appropriate templates for both groups were used to derive default anatomical regions^30^, which, along with the default electrode locations, were entered into the forward boundary element head model (BEM) provided by the openMEEG algorithm^63^. The tissue conductivity values were those used in O’Reilly et al.^30^ in 7-months-old infants (0.33 S/m for the grey matter, 0.0041 S/m for the skull, and 0.33 S/m for the scalp).

For the modeling of time-varying source signals, (current density) of all cortical voxels, a minimum norm estimate inverse solution was applied after appropriate noise covariance estimation. Matrices were then scaled through dynamical Statistical Parametric Mapping normalization^64^. Averaging current density across voxels yielded time series for 62 cortical areas (region of interest ROIs), defined by the standardized parcellation scheme introduced by Desikan and Killiany^65^. Finally, for all ROIs, we extracted the average signal for the corresponding ERP peak windows defined through the cluster-based statistic, separately for each SOUND DURATION (short, long), STIMULUS TYPE (tones, noise), and AGE GROUP (4, 9 months). We then applied nonparametric permutation testing on cortical source activity maps. Specifically, we compared long vs. short stimuli separately for tones and noises and for each age group through a paired t-test (*p* < 0.05). STIMULUS DURATION labels were permuted 500 times through the Monte Carlo method to create the histogram of the test statistic. Time windows of interest were defined by referring to points differentiating the two age groups after the application of the cluster-based statistic.

## Data Availability

Data is available on request (s.polver@campus.unimib.it) due to privacy and ethical restrictions.
